# The Book World of Medicine and Science

**Published:** 1898-11-12

**Authors:** 


### THE BOOK WORLD OF MEDICINE AND SCIENCE.

Skiagraphic Atlas. By John Poland, F.R.C.S. (London: Smith, Elder, and Co. 1898. Pp. 40. 20 illustrations. 5s.)

It is undoubtedly a fact that the insight which the Röntgen rays give into the hidden facts of developmental osteology, will ere long radically alter our preconceived ideas. The work before us brings this matter out most clearly, and adds to our knowledge of the ossification of the lower ends of the bones of the fore-arm, carpus, and hand to a considerable degree. The book consists of twenty illustrations, nineteen of which are skiagrams, skilfully taken by Mr. William Webster (except one), and thoroughly well reproduced. These represent the bones at various ages, ranging from one to seventeen years, and the gradual process of development is particularly well and faithfully pourtrayed. It would, perhaps, have been well if the *aspect* from which the bones are viewed had been stated. The author has prefixed his own account of the anatomy and ossification of the several bones, but there is one statement in this which does not agree with the fact as it is brought out by the skiagram facing page 8, namely, that the centre of ossification for the lower epiphysis of the radius is well marked at the end of the *first* year. In the letterpress, however, Mr. Poland says, "Towards the end of the *second* year the osseous nucleus of the lower epiphysis appears." It would be interesting to learn whether other skiagrams taken at the same age confirm or not that depicted in this work, showing the much earlier commencement of this ossification. Another suggestion that these skiagrams appear to show is that ossification is later in the cases of children who are born late in the family than in those who come first. It would need, however, a very extended investigation to prove the truth of this, but it is interesting in connection with the fact that rickets seem to attack the last members of a family rather than the earlier ones. There is an error in the letterpress under the second skiagram after p. 16—the word "following" should be "preceding." We have nothing but praise for this work, and can only hope that the author will bring out other atlases of like nature and worth.

Conservative Gynecology and Electro-Therapeutics: A Practical Treatise on the Diseases of Women and their Treatment by Electricity. Third edition, re-written and greatly enlarged. By G. Betton Massey, M.D., Physician to the Gynecic Department of Howard Hospital, Philadelphia. Illustrated with twelve chromo-lithographic plates, numerous plates of photographs, and other engravings in the text. (The F. A. Davis Co., publishers, Philadelphia. Pp. 400. 3.50 dol. net.)

In this third edition the author has considerably extended the scope of his work. The second edition was issued under the more modest title of "Electricity in the Diseases of Women," but as the advocate of electricity commonly discovers that it is applicable to most if not all pathological conditions, this transition is perhaps a natural one. There is much to commend in this book, and much that commands assent. The style is specially clear, and the author's way of regarding the sequence of events in gynecological pathology is generally a true one. As a handbook to the methods of using electricity in medicine the work will be found complete and accurate, and therefore useful. The weakness of the work is derived from the fact that it claims too much for electricity; and while the physician who has just bought a battery, and who is seeking opportunities to use it, will find encouragement to try it in nearly every case he meets, the philosophic doctor will recognise that the very universality of the claim places electricity among the "faith cures." If the author had restricted its scope, the true and scientifically established action of electricity in certain cases would have received a greater recommendation. But he has, in our opinion, further weakened his position by the "basis" on which he places "the medicinal value of electricity" (p. 26). This basis is the assumption, which he quotes from a paper by Professor Dolbear, that the molecular activity of cells is mainly "ultimately electric." Needless to say, this assumption is unfounded. But we may leave this point, as being mainly of academic interest, and turn to one or two matters of practical importance. The first is the statement made on p. 28, and repeated on pp. 29 and 30, that electrical treatment can do no harm "even in unaccustomed hands." This may be true if the dosage be very weak; but in that case the treatment may be considered as on a par with bread pills, which also "do no harm." If electricity is to be regarded (as we think it is entitled to be) as something more than a symbol for the exercise of faith, it must be administered in such doses that its injudicious or ignorant use may be productive of harm. In the second place, we must enter a strong protest against the recommendation that electricity should be used in the treatment of ectopic gestation. He says (p. 215): "During these early months (*i.e.*, till the end of the fourth month) the electric treatment is clearly the method of choice." On the contrary, it has been repeatedly demonstrated that when ectopic gestation exists the patient's life is not safe until the gravid tube has been dealt with surgically.

The Wanton Mutilation of Animals. By G. Fleming, C.B., LL.D. (London: G. Bell and Sons. 1898. Pp. 24. Folio. Illustrated. Price 2s. 6d.)

This essay, which appeared in the *Nineteenth Century* a year or two ago, has been most opportunely reprinted at the request of the R.S.P.C.A. No one in this country can speak with greater force and authority on the chief subjects of this essay—the cropping and docking of horses—than Dr. Fleming, on whose recommendation, as is well known, the War Office officially condemned these wanton practices. The custom of docking horses, barbarous and utterly useless as it is, has nothing but fashion to recommend it. Yet its observance has been elevated by horse breeders into something like a fetish. Cropping, happily, has fallen into desuetude. Let us hope docking will follow it. Anyone open to reason must surely be convinced by Dr. Fleming's temperate and cogent arguments of its wantonness and folly. But we suppose "conscientious objectors" will still be found to insist on its perpetration—"conscientious objectors" who will be convinced by nothing less than a change of fashion and threatened pecuniary loss. But barbarities other than docking are also condemned in these pages by Dr. Fleming—the worming, cropping, and docking of dogs, the dubbing of game fowl. Dr. Fleming gives a very interesting historical survey of his subject, and illustrates it by some excellent reproductions of old pictures. That this publication may strengthen the hands of the R.S.P.C.A. is, we feel sure, the sincere desire of every true lover of animals.

